# Dietary Diversity and Nutritional Status Among Rwandan Women Engaged in Agriculture: A Cross‐Sectional Study

**DOI:** 10.1002/puh2.214

**Published:** 2024-07-08

**Authors:** Sunday François Xavier, Philemon Kwizera, Yves Didier Umwungerimwiza, Rutayisire Reverien, Kanimba Philbert, Ilinde Niyigena Delice, Maryse Umugwaneza

**Affiliations:** ^1^ Department of Human Nutrition and Dietetics School of Public Health College of Medicine and Health Sciences University of Rwanda Kigali Rwanda; ^2^ Department of Medical Imaging Sciences School of Health Sciences College of Medicine and Health Sciences University of Rwanda Kigali Rwanda; ^3^ Department of Biomedical Laboratory Sciences School of Health Sciences College of Medicine and Health Sciences University of Rwanda Kigali Rwanda

**Keywords:** agriculture, dietary diversity, nutritional status, Rwanda, women

## Abstract

**Background:**

Dietary diversity is crucial for nutritional adequacy, particularly among women of reproductive age who have increased nutritional needs due to menstruation, pregnancy, and lactation. This is especially important in addressing anemia in Rwanda, which poses significant health risks for both mothers and children. This study assessed the dietary diversity, nutritional status, and related factors among Rwandan women engaged in agriculture.

**Methods:**

In 2022, a cross‐sectional study in Nyamagabe, Karongi, and Nyabihu districts, Rwanda, focused on high malnutrition rates. Agriculture households with children under 5 were sampled using a multistage method. Data, including nutritional status via MUAC and dietary diversity via 24‐h recall, were collected digitally through Kobo Collect.

**Results:**

The study included 439 respondents, with a mean age of 33 years. Among participants, 359 (81.8%) had low dietary diversity, with Nyamagabe having the highest proportion at 39%. Anemia prevalence was 22.1%, with Karongi having the highest at 10.7%. Factors associated with higher odds of high dietary diversity included the education of the household head (adjusted OR = 6.4, 95% CI: 1.05–39.7), age of women (adjusted OR = 3.03, 95% CI: 1.1–7.8), and wealth status (adjusted OR = 1.66, 95% CI: 0.51–5.4). Conversely, the occupation of women (adjusted OR = 0.13, 95% CI: 0.001–0.19), reading skills (adjusted OR = 0.27, 95% CI: 0.1–0.72), and family size (adjusted OR = 0.63, 95% CI: 0.35–1.1) were associated with lower odds of lower dietary diversity.

**Conclusion:**

The findings highlight a significant nutritional challenge among Rwandan women, with low dietary diversity, significant rates of anemia, and food insecurity. The study calls for an urgent need for targeted nutritional interventions to improve dietary diversity and address micronutrient deficiencies among women in agriculture to enhance maternal health and child development, thereby contributing to broader public health goals.

## Graphical abstract

The study included 439 respondents among the participants; overall, 359 (81.8%) had low dietary diversity. Factors associated with higher odds of high dietary diversity included the education of the household head, age of women, and wealth status. Conversely, the occupation of women, reading skills, and family size were associated with lower odds of lower dietary diversity.

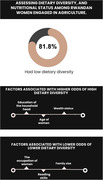

## 1 Background

Rwanda boasts a variety of agricultural production systems distributed across its different agro‐ecological zones [1]. The population is primarily rural, with 72.1% residing in rural areas. Females slightly outnumber males, making up 51.5% of the population compared to 48.5% of males [2]. Approximately 2.3 million Rwandan households, constituting 69% of private households, engage in agricultural activities, with 63% focusing on crop farming. Key crops include beans, maize, cassava, sweet potato, and banana, with nearly half cultivating at least one fruit type [2]. The prevalence of malnutrition among rural dwellers, particularly women and children, has plateaued despite focused government efforts. The primary causes include insufficient nutritional intake and stagnant improvements in household food security [3]. Despite farming being common in rural areas, past research suggests limited knowledge about small‐scale vegetable farming and healthy eating. This situation leads to low dietary diversity, with households relying on starch‐based meals readily available in the community [4].

Dietary diversity is an essential indicator of nutritional adequacy and reflects the variety of foods consumed within a given period [5]. It is associated with favorable health outcomes, including a reduced risk of micronutrient deficiencies, improved maternal health, and enhanced child development [6]. Rural women in the reproductive age group experience heightened nutritional vulnerability due to the substantial physiological demands imposed by their reproductive functions. Within this age bracket, women undergo various critical phases, including menstruation, pregnancy, and lactation, each of which necessitates an augmented intake of essential nutrients to adequately support both their health and the well‐being of their offspring during these critical life stages [7].

The minimum dietary diversity (MDD) for women indicator is a recent tool that can be employed to assess the diversity and quality of women's diets [8]. This indicator emphasizes the importance of consuming a variety of foods or food groups over time. A diverse diet is beneficial because different foods provide essential nutrients, such as proteins, carbohydrates, fats, vitamins, and minerals, all necessary for the body's optimal function. This indicator calculates the nutritional sufficiency of women using 10 major dietary groups. This list includes grains, white root tubers, plantains, pulses, nuts and seeds, dairy, meat and fish, eggs, dark green leafy vegetables, vitamin A–rich fruits and vegetables, and others [9]. Women who consume five or more food groups are more likely to meet their needs for essential vitamins and minerals, which ensures they receive the nutrients required to maintain good health [10]. Conversely, women who consume foods from fewer than five groups are at a higher risk of micronutrient deficiencies [11]. Scientific research suggests that the dietary diversity score can indicate household food security and the adequacy of micronutrient and macronutrient intake in women of reproductive age, which is crucial for combating anemia [12].

Anemia represents a significant public health challenge in developing nations such as Rwanda and can lead to various complications for pregnant women and their unborn children. These complications encompass issues like premature births, low birth weight, postpartum difficulties such as excessive bleeding and susceptibility to infections, as well as fatigue due to body weakness. Additionally, anemia can also result in adverse outcomes for the fetus, including inadequate intrauterine growth and delayed developmental progress [13]. According to Rwanda's demography health survey report, approximately 13% of women aged 15–49 suffer from anemia. Of this group, 9% have mild anemia, 4% have moderate anemia, and 1% have severe anemia [14].

Within this context, this study aimed to determine dietary diversity and its association with nutritional and anthropometric status, particularly focusing on iron, among women engaged in agriculture in Rwanda.

## 2 Methods

### 2.1 Study Design and Settings

**FIGURE 1:**
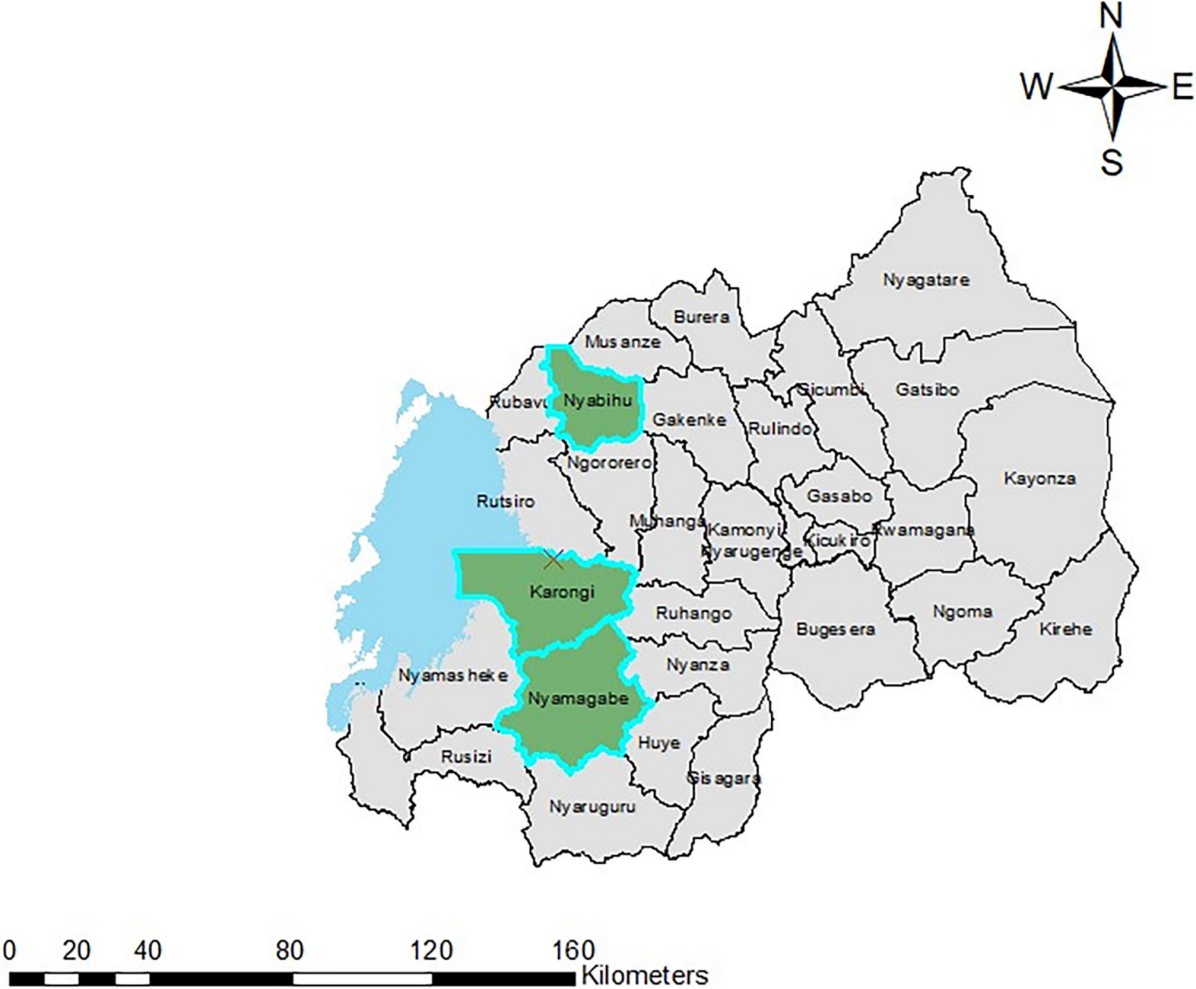
Map of study sites in Nyabihu, Karongi, and Nyamagabe districts for assessing the dietary diversity and nutritional status of Rwandan women in agriculture in 2022.

This cross‐sectional study was conducted in 2022 in the western region of Rwanda, specifically focusing on three districts: Nyamagabe, Karongi, and Nyabihu (Figure 1). These districts were purposively selected based on their notable prevalence of malnutrition among children under 5 years old, as documented in the Rwandan Demographic and Health Survey [14]. In Karongi, cluster one focused on subsistence farming, with an area of 980 km^2^ and a population of 373,869. The district has 13 sectors, of which 4 were randomly selected to host the study [15]. In Nyabihu, cluster two, known for Irish potato farming, covered a mountainous area of 531.6 km^2^ with a population of 294,749 [16]. Nyamagabe, the location of cluster three, is a tea‐growing region with an area of 1090 km^2^ and a population of 371,501. Agriculture is the primary livelihood in these districts, with various crops being cultivated [17].

### 2.2 Study Population and Sampling

Selecting districts, sectors, villages, and participating households followed a multistage sampling approach. Initially, cluster sampling identified three districts based on specific farming practices: Nyamagabe (tea crop cultivation), Karongi (subsistence farming), and Nyabihu (Irish potato farming). Villages within each district were randomly selected using computer‐based methods of rand between functions. The sample size was calculated for proportions, including 10% for potential nonresponses. In each selected village, approximately four households, each having a child under 5 years of age and engaged in agriculture, were selected to participate in the study. Community health workers (CHWs) provided lists of eligible households from which systematic sampling identified participants. Caregivers of children aged under 5 from these households were the study participants.

### 2.3 Study Instrument and Data Collection

The data collection tool was a questionnaire created by amalgamating questions from validated questionnaires used in previous studies [18] and dietary‐related inquiries adapted from the Food and Agriculture Organization of the United Nations (FAO) 2016 guidelines [19]. The tool was digitally administered via Kobo Collect on a tablet. Before deployment, the questionnaire underwent a pilot phase to ensure its proper display, clarity of questions for the participants, adequacy in gathering the required information, and adherence to ethical considerations, including time constraints.

Enumerators briefed the selected participants or caregivers of children under 5 years in each household about the study during the enumeration process. These interviews were conducted face‐to‐face within the households using the questionnaire mentioned above. CHWs facilitated the process by helping to identify selected households. The data were recorded using an electronic tablet, and later, after completing the daily surveys, the enumerator uploaded the collected data to ensure easy access for the data manager.

### 2.4 Assessment of the Nutritional Status of Women

The measurement of mid‐upper arm circumference (MUAC) serves as a useful indicator for assessing the nutritional status of women [20]. MUAC is frequently employed as a gauge of fat‐free mass. This investigation chose it as a surrogate for body weight due to its independence from gestational age effects [21]. The measurement of MUAC was carried out using a nonstretchable MUAC tape with a precision of 0.1 cm. Instances where MUAC values fell below 25.0 cm were considered indicative of low body weight in the analytical assessments. Notably, establishing universally accepted MUAC cutoff points needs to be improved, underscoring the need for further consensus in this area [22].

### 2.5 Measurements of the Household Dietary Diversity

The measurement of the household dietary diversity used the 24‐h recall strategy, where the child caretaker was asked to recall what the household had consumed over 24 h. The dietary diversity questionnaire has 10 different food groups based on their nutrients: (1) grains, white root, tubers, and plantains; (2) pulses (beans, peas, and lentils); (3) nuts and seeds; (4) dairy; (5) meat and fish (poultry and fish); (6) eggs; (7) dark green leafy vegetables; (8) vitamin A–rich fruits and vegetables; (9) other vegetables; and (10) other fruits. Each group is assigned a score of “0” or “1” based on consumption; a household is given a score of 1 for each food group consumed for 24 h. The scores are then summed up to obtain the Household Dietary Diversity Score (HDDS), a higher score reflecting greater dietary diversity. The score may range from 0 to 10. Then, households are put into low DD and high DD.

### 2.6 Measurements of the Household Food Security

The methodology for assessing households’ food security involved using the Household Food Insecurity Access Scale (HFIAS) provided by the Food and Nutrition Technical Assistance Communities (FANTA) [18]. This measurement aimed to determine the food security status of the households and identify associated factors. The HFIAS assesses the household's food insecurity over 4 weeks (approximately 30 days). The calculation implied summing up the codes for each frequency of occurrence question, with codes assigned to cases where the response to occurrence questions was “No” (e.g., if Q1 = 0, then Q1a = 0, if Q2 = 0, then Q2a = 0).

The total score could range from 0 to 27. A score of 27 indicated that a household had responded “often” to all nine frequency questions, signifying high food insecurity of access. Conversely, a score of 0 would result if the household responded no to all occurrence questions, indicating a lower level of food insecurity. To provide a more precise assessment of food security and insecurity, the households were categorized based on their scores. The recommended categories of FANTA were as follows: 0–1 (food secure), 2–8 (mild food insecure), 9–16 (moderate food insecure), and 17–27 (severe food insecure) [18]. Subsequently, the study calculated and reported the frequency and percentage for each category. The data were further analyzed by merging the three categories of food insecurity into two broad categories, comprising food security and food insecurity, to simplify the analysis and reporting.

### 2.7 Measurement of Blood Anemia

Blood was tested for anemia using a HEMOCUE device. Finger capillary blood was obtained via a lancet, with a careful aseptic technique. A second drop of blood was collected with a capillary transfer tube for analysis. Anemia was determined with hemoglobin levels <12 g/dL for mothers and <11 g/dL for children [23].

### 2.8 Data Analysis

The data were generally collected using digitalized tools and then sent to the data manager daily. Laboratory findings were also added to the primary data set after laboratory analysis for anemia was done. Data were downloaded from the Kobo toolbox in Excel format, checked for completeness, and then imported into SPSS V25 for coding and analysis. Descriptive statistics, including frequencies and percentages, were computed to summarize the central tendencies and variability of the investigative variables. The associations between demographic data and dietary diversity were conducted using the chi‐square or Fisher exact tests where necessary. The significance level for all analyses was set at 0.05.

### 2.9 Ethical Considerations

The study received ethical clearance from the University of Rwanda Institutional Review Board with Ethics approval Notice: No. 259/CMHS IRB 2021 and approval from the National Institute of Statistics (No. 0305/2021/10/NISR). Authorization to collect field data was granted by the Ministry of Local Governance (No. 0806/07.01). Participants were informed about the study's design, objectives, and importance, and individual informed consent was obtained after assuring confidentiality to participants. Participation was voluntary, data were anonymized to protect privacy, and findings were reported honestly.

## 3 Results

### 3.1 Sociodemographic and Other Related Characteristics of Women Engaged in Agriculture

The study surveyed 439 respondents with a mean age of 33 years. The details of the basic sociodemographic characteristics are shown in Table 1.

**TABLE 1:** Sociodemographic characteristics of Rwandan women in agriculture, southern and western regions, 2022.

	Total	Karongi	Nyabihu	Nyamagabe
Variables	*n* (%)	*n* (%)	*n* (%)	*n* (%)
Age of the mothers (years)				
17–24	52 (11.8)	22 (4.8)	16 (3.7)	15 (3.4)
25–34	201 (45.8)	78 (17.7)	63 (14.4)	58 (13.3)
35 and above	186 (83.6)	50 (11.2)	56 (12.8)	81 (18.6)
Mothers’ marital status				
Live with partner	376 (85.6)	122 (32.4)	127 (33.8)	127 (33.8)
Do not live with Partner	63 (14.4)	28 (44.4)	8 (12.7)	27 (42.9)
Type of education attended				
No education	4 (0.9)	0 (0.0)	1 (25)	3 (75)
Lower education	253 (57.6)	81 (32)	68 (37.9)	104 (41.1)
Higher education	182 (41.5)	69 (37.9)	66 (36.3)	47 (25.8)
Know how to read and write	357 (81.3)	128 (85.3)	107 (79.3)	120 (77.9)
Mothers’ employment activities beyond their agricultural work				
Casual labor	66 (15)	15 (22.7)	13 (19.7)	38 (57.6)
Petty trade and or handcrafts	9 (2.1)	2 (22.2)	6 (66.7)	1 (11.1)
Monthly income	4 (0.9)	3 (75)	0 (0)	1 (25)
Total	79 (18)	20 (4.56)	19 (4.33)	40 (9.11)
Head of household				
Husband	367 (83.6)	116 (31.6)	125 (34.1)	126 (34.3)
Wife	72 (16.4)	34 (47.2)	10 (13.9)	28 (38.9)
Age of household head in years				
21–45	352 (80.2)	123 (34.8)	119 (33.7)	111 (31.4)
46–69	81 (18.5)	25 (31.3)	15 (18.8)	40 (50)
70–92	6 (1.4)	2 (33.3)	1 (16.7)	3 (50)
Education of the head of household				
No formal education	58 (13.2)	18 (31)	14 (24.1)	26 (44.8)
Primary education	270 (61.5)	91 (33.7)	68 (25.2)	111 (41.1)
Secondary and above education	111 (25.3)	41 (36.9)	53 (47.7)	17 (15.3)
Marital status of the head of household				
Live with partner	388 (88.4)	133 (34.3)	130 (33.5)	125 (32.2)
Do not live with partner	51 (11.6)	17 (33.3)	5 (9.8)	29 (56.9)
Family size				
0–3	78 (17.8)	25 (32.1)	19 (24.4)	34 (43.6)
4–6	248 (56.5)	86 (34.7)	72 (29)	90 (36.3)
>6	113 (25.7)	39 (34.5)	44 (38.9)	30 (26.5)
Household food security^a^				
Food secure	28 (6.4)	11 (39.3)	10 (35.7)	7 (25)
Food insecure	411 (93.6)	139 (33.8)	125 (30.4)	147 (35.8)
Wealth category of the household^b^				
Category 1 (abject poverty)	29 (6.6)	13 (44.8)	11 (37.9)	5 (17.2)
Category 2 (very poor)	223 (50.8)	74 (33.2)	85 (38.1)	64 (28.7)
Category 3 (poor)	186 (42.4)	63 (33.9)	38 (20.4)	85 (45.7)
Category 4 (resourceful poor)	1 (0.2)	0 (0)	1 (100)	0 (0)

^a^ Household food security was calculated using the Household Food Insecurity Access Scale (HFIAS) provided by the Food and Nutrition Technical Assistance Communities [18].

^b^ The Wealth Category of the household, known as Ubudehe, was classified based on the household's socio‐economic activities, following the categorization established by the Rwandan government.

### 3.2 Food Consumption Patterns of Women Engaged in Agriculture

**FIGURE 2:**
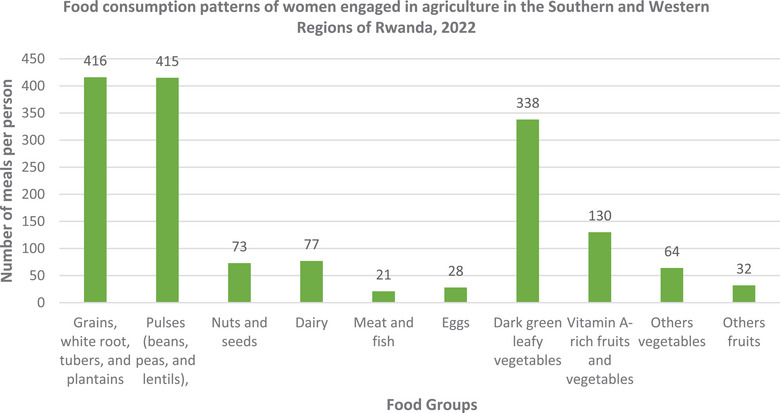
Food consumption patterns of women engaged in agriculture in the southern and western regions of Rwanda, 2022 (*n* = 439).

Figure 2 shows the food groups that women consumed in the last 24 h; a high number (416) included grains, white root vegetables, tubers, and plantains in their diets. This was closely followed by those who consumed pulses (415). On the other hand, eggs were consumed by the fewest women (28), with meat and fish being the next lowest at 21 (Figure 2).

### 3.3 Nutritional Status and Blood Biochemistry Among Rwandan Women Engaged in Agriculture in the Southern and Western Regions

The findings summarized in Table 2 showed that 97.3% of mothers had an MUAC measurement above 22 cm and 2.7% had a measurement less than 22 cm, where the majority obtained in Nyamagabe (83.3%), whereas 12 (2.7%) of the mothers were underweight, whereas Karongi and Nyamagabe had the same proportion with 6 participants and 80 (19.4%) were overweight. Furthermore, 359 (81.8%) participants had low dietary diversity where Nyamagabe had the highest proportion with 39%.

**TABLE 2:** Assessment of the nutritional status and Blood Biochemistry among Rwandan women engaged in agriculture in the southern and western regions of Rwanda, 2022.

Variables	*n* (%)	Karongi	Nyabihu	Nyamagabe
MUAC (cm)				
<22	12 (2.7%)	0 (0.0%)	2 (16.7%)	10 (83.3%)
>22	427 (97.3%)	150 (35.1%)	133 (31.1%)	144 (33.7%)
BMI (indices)				
Normal	303 (69%)	116 (38.3%)	74 (24.4%)	113 (37.3%)
Obesity	39 (8.9%)	9 (23.1%)	27 (69.2%)	3 (7.7%)
Overweight	85 (19.4%)	19 (22.4)	34 (40%)	32 (37.6%)
Underweight	12 (2.7%)	6 (50%)	0 (0.0%)	6 (50%)
Hemoglobin (g/dL)				
Normal	269 (92.12%)	109 (40.5%)	48 (17.8%)	112 (41.6%)
Mild anemia	21 (7.2%)	12 (57.1%)	2 (9.5%)	7 (33.3%)
Severe anemia	2 (0.7%)	1 (50%)	0 (0%)	1 (50%)
Dietary diversity score				
Low dietary diversity	359 (81.8%)	131 (36.5%)	88 (24.5%)	140 (39%)
High dietary diversity	80 (18.2%)	19 (23.8%)	47 (58.8%)	14 (17.5%)

*Note*: MUAC, mid‐upper arm circumference, uses a tape to assess nutritional status (children and pregnant women: circumference in cm of the upper arm at the midpoint between the shoulder and the elbow); BMI, body mass index (*w* (kg)/*H* (m)^2^); Hb, hemoglobin as an indication of protein in red blood cells (grams per deciliter (g/dL) of blood); DD, dietary diversity: number of food groups consumed in 24 h.

### 3.4 Sociodemographic Characteristics and MDD Distribution

Table 3 presents the sociodemographic characteristics and distribution of MDD scores among Rwandan women engaged in agriculture in 2022. A total of 439 participants were included, with 81.8% having an MDD score <5 and 18.2% >5. Age distribution showed significant differences (*p* = 0.05), with higher proportions of younger participants (17–24 years) in the >5 MDD group. Literacy significantly affected MDD scores (*p* = 0.005), with higher rates among those who could read and write (92.5%); in addition, Employment type also influenced MDD scores (*p* = 0.002).

**TABLE 3:** Association between minimum dietary diversity (MDD) with sociodemographic characteristics (*n* = 439) among Rwandan women engaged in agriculture in the southern and western regions of Rwanda, 2022.

Variable	Total (*n* = 439)	DD score <5 (*n* = 359)	DD score >5 (*n* = 80)	*p* value
Number of participants	439 (100)	359 (81.8)	80 (18.2)	
Age of women, years, *n* (%)				0.05
17–24	52 (11.8)	37 (71.2)	15 (28.8)	
25–34	201 (45.8)	163 (81.1)	38 (18.9)	
35 and above	186 (42.4)	159 (85.5)	27 (14.5)	
Head of household, *n* (%)				0.440
Husband	367 (83.6)	301 (82)	66 (18)	
Wife	72 (16.4)	58 (80.6)	14 (19.4)	
Age of household head, years, *n* (%)				0.601
Less than 30	68 (15.5)	59 (86.8)	9 (13.2)	
30–39	186 (42.4)	153 (82.3)	33 (17.7)	
40–59	164 (37.4)	130 (79.3)	34 (20.7)	
60 and above	21 (4.8)	17 (81)	4 (19)	
Marital status, *n* (%)				0.695
Legally married	246 (56)	200 (81.3)	46 (18.7)	
Widow	6 (1.4)	6 (100)	0 (0.0)	
Legally divorced	3 (0.7)	3 (100)	0 (0.0)	
Single mother	54 (12.3)	45 (83.3)	9 (16.7)	
Cohabitation	130 (29.6)	105 (80.8)	25 (19.2)	
Know how to read and write, *n* (%)				0.005
Yes	357 (81.3)	283 (78.8)	74 (92.5)	
No	82 (18.7)	76 (21.2)	6 (7.5)	
Mothers’ employment activities beyond their agricultural work, *n* (%)				0.002
Employment with monthly income	4 (0.9)	1 (0.3)	3 (3.8)	
Petty trade and or handcrafts	9 (2.1)	6 (1.7)	3 (3.8)	
Casual labor	66 (15)	62 (17.3)	4 (5)	
Head of household education, *n* (%)				0.619
Tertiary education	7 (1.6)	4 (57.1)	3 (42.9)	
Not attended any	52 (11.8)	44 (84.6)	8 (15.4)	
Didn't finish primary	270 (61.5)	222 (82.2)	48 (17.8)	
Did not finish secondary	47 (10.7)	39 (83)	8 (17)	
Finished primary	6 (1.4)	5 (83.3)	1 (16.7)	
Completed secondary	57 (13)	45 (78.9)	12 (21.1)	
Family size, *n* (%)				
0–3	78 (17.8)	64 (82.1)	14 (17.9)	0.291
4–6	248 (56.5)	208 (83.9)	40 (16.1)	
>6	113 (25.7)	87 (77)	26 (23)	
Wealth category, *n* (%)				0.687
Category 1 (abject poverty)	223 (50.8)	178 (79.8)	45 (20.2)	
Category 2 (very poor)	1 (0.2)	1 (100)	0 (0)	
Category 3 (poor)	29 (6.6)	25 (86.2)	4 (13.8)	
Category 4 (resourceful poor)	186 (42.4)	155 (83.3)	31 (16.7)	

### 3.5 Predictors of Inadequate Dietary Diversity Among Rwandan Women in Agriculture (southern and western regions, 2022)

Table 4 examines factors influencing inadequate dietary diversity (DD score <5) in Rwandan women in agriculture. Of the 439 participants, 81.8% had insufficient diversity. Younger age (under 24) correlated with better diversity (28.8%). Marital status, literacy, employment type, household head's education, family size, and wealth category also impacted dietary diversity.

**TABLE 4:** Predictors of inadequate dietary diversity among Rwandan women engaged in agriculture in the southern and western regions of Rwanda, 2022.

Variable	DD score <5 (*n* (%))	DD score > 5 (*n* (%))	OR (95% CI)	Adjusted OR (95% CI)
Number of participants	359 (81.8)	80 (18.2)		
Age, years, *n* (%)				
17–24	37 (71.2)	15 (28.8)	1	1
25–34	163 (81.1)	38 (18.9)	0.57 (0.28–1.15)	3.03 (1.1–7.8)
35 and above	159 (85.5)	27 (14.5)	0.41 (0.2–0.86)	1.22 (0.65–2.3)
Head of household, *n* (%)				
Husband	301 (82)	66 (18)	1	1
Wife	58 (80.6)	14 (19.4)	0.68 (0.334–1.402)	0.73 (0.33–1.5)
Marital status, *n* (%)				
Legally married	200 (81.3)	46 (18.7)	1	1
Widow	6 (100)	0 (0.0)	1 (0.6–1.7)	0.78 (0.43–1.4)
Legally divorced	3 (100)	0 (0.0)	0.87 (0.39–1.9)	0.48 (0.16–1.3)
Single mother	45 (83.3)	9 (16.7)	0	0
Cohabitation	105 (80.8)	25 (19.2)	0	0
Mothers know to read and write, *n* (%)	76 (21.2)	6 (7.5)	0.3 (0.1–0.7)	0.27 (0.1–0.72)
Mothers’ employment activities beyond their agricultural work, *n* (%)				
Employment with monthly income	1 (0.3)	3 (3.8)	1	1
Petty trade and/or handcrafts	6 (1.7)	3 (3.8)	0.2 (0.05–0.9)	0.6 (0.004–0.8)
Casual labor	62 (17.3)	4 (5)	1.8 (0.3–9.8)	0.04 (0.004–0.5)
Education of the head of household, *n* (%)				
Not attended any	44 (84.6)	8 (15.4)	0.24 (0.45–1.29)	6.4 (1.05–39.7)
Didn't finish the primary	222 (82.2)	48 (17.8)	0.35 (0.7–1.8)	3.1 (0.23–43.2)
Finished primary	5 (83.3)	1 (16.7)	0.62 (0.62–1.31)	0.91 (0.31–2.6)
Didn't finish secondary	39 (83)	8 (17)	0.26 (0.19–3.65)	0.9 (0.45–2.1)
Completed secondary	45 (78.9)	12 (21.1)	0.75 (0)	0
Tertiary education	4 (57.1)	3 (42.9)	1	1
Family size, *n* (%)				
0–3	64 (82.1)	14 (17.9)	1	1
4–6	208 (83.9)	40 (16.1)	1.36 (0.66–2.82)	0.63 (0.35–1.1)
>6	87 (77)	26 (23)	0.87 (0.45–1.7)	0.72 (0.3–1.6)
Household wealth category, *n* (%)				
Category 1 (abject poverty)	25 (86.2)	4 (13.8)	1	1
Category 2 (very poor)	178 (79.8)	45 (20.2)	1.58 (0.52–4.77)	1.66 (0.51–5.4)
Category 3 (poor)	155 (83.3)	31 (16.7)	1.25 (0.4–3.84)	1.1 (0.3–3.9)
Category 4 (resourceful poor)	1 (100)	0 (0)	0	0

## 4 Discussion

The findings of this study indicated significant correlations between MDD and several factors, including respondents’ age, their husbands’ educational levels, family monthly income, and occupational status. There was a noteworthy association between the respondents’ employment status and their literacy levels.

According to this study, the average dietary diversity for women was low, with a majority consuming an average of three food groups per day. Only 18.2% of women achieved the recommended intake of five or more food groups, which contrasts with findings from similar studies in rural Tanzania with 10% [24]. These findings are also lower compared to other studies: Jeldu District, West Shoa Zone, Oromia Ethiopia, 81.9% [25], Burkina Faso, 31% [26], two districts of Southern Benin, 41% [27], South Africa, 25% [28], India, 46.2% [29], Algeria, 32% [30], and Bangladesh, 65% [31]. Variations in the study period, such as the season of data collection, geographical location, and socio‐cultural factors, could account for these disparities.

Furthermore, the research indicated that the majority of women's diets consisted of starchy staples, with legumes, particularly beans, being the second most consumed food group. A striking 95.4% of women reported consuming carbohydrate‐rich foods such as grains, white root vegetables, tubers, and plantains. This finding aligns with similar studies conducted in Ethiopia [25], Ghana [32], Zambia [33], and Kenya [34], emphasizing a common dietary pattern across diverse geographical regions.

The result of multivariable logistic regression analysis, detailed in Table 4, revealed that women who were literate were twice as likely to achieve adequate dietary diversity compared to those who were illiterate. This finding resonates with previous studies conducted in Ataye District, North Shoa Zone, Central Ethiopia [35], suggesting that educated mothers may benefit from increased access to healthcare services, thereby gaining better awareness of diverse food options. They may also have a better grasp of the importance of increasing dietary variety and meal frequency, contrasting with less educated mothers, who may face limitations in accessing a wider variety of food groups.

This study identified a significant correlation between MDD and family size among Rwandan women engaged in agriculture. The likelihood of achieving a higher MDD score was more than double for women with fewer than three family members compared to those with more than three members, that is, larger families. This finding is reminiscent of a study conducted in Eastern Ethiopia, where the odds of achieving a higher MDD score for women with smaller families were more than seven times greater than those with larger families [36]. However, this outcome contradicts the results of research conducted in Bangladesh [37], suggesting that the number of dependents in larger families may impact dietary diversity differently, potentially reflecting variations in household socioeconomic status.

In summary, this analysis of dietary diversity and nutritional status among Rwandan women engaged in agriculture in Rwanda reveals that the typical diet has low diversity, likely contributing to micronutrient deficiencies such as low iron. This was confirmed by a low hemoglobin level in this group of women, with 7.2% of participants having mild or moderate anemia of the women falling below the threshold hemoglobin sufficiency of 11.9–15.0 g/dL [38].

## 5 Limitations

The study was limited in several ways. First, even though women consumed several meals high in micronutrients at a moderate frequency, the study should have considered the amount of food they consumed, making it hard to assess whether their diet satisfied nutritional needs. One notable constraint of the study is its incomplete coverage of Rwanda's seasons, which may have resulted in the omission of seasonal fluctuations in food supply and consumption patterns.

Nonetheless, successful techniques used to lessen the research findings’ limits included using objective indicators like biomarkers or nutritional evaluations, validated instruments, cutting the recollection period, and triangulating data.

## 6 Conclusion

The study found that dietary diversity was low throughout the study. Given the importance of dietary diversity in nutrition and health, there must be increased efforts to promote access to and awareness of diverse and appropriate nutrition. Promoting a diversified diet and managing anemia are essential to improving well‐being. The study noted the need to increase access to different foods, create work possibilities for women, improve family planning, and provide nutrition education. Future studies should assess the efficacy of specific nutritional interventions and educational activities.

## Author Contributions

S.F.X. contributed to study design, funding acquisition, ethical submissions, and supervision of data collection, methodology writing, and manuscript review. P.K. contributed to data analysis and manuscript writing. Y.D.U. contributed to manuscript writing. R.R. contributed to data management and analysis. I.N.D. contributed to data analysis. K.P. contributed to blood sample collection and analysis. M.U. contributed to guiding the project.

## Conflicts of Interest

The authors declare no conflicts of interest.

## Data Availability

The data that support the findings of this study are available from the corresponding author upon reasonable request.
